# Influence of Mix Design Parameters on Fresh and Hardened Properties of Geopolymer Concrete: A State-of-the-Art Review

**DOI:** 10.3390/polym18070854

**Published:** 2026-03-31

**Authors:** Seemab Tayyab, Wahid Ferdous, Weena Lokuge, Tuan Ngo, Andreas Gerdes, Allan Manalo

**Affiliations:** 1 Centre for Future Materials (CFM), School of Science, Engineering and Digital Technologies, University of Southern Queensland, Toowoomba, QLD 4350, Australia; seemab.tayyab@unisq.edu.au (S.T.); weena.lokuge@unisq.edu.au (W.L.); allan.manalo@unisq.edu.au (A.M.); 2 Department of Infrastructure Engineering, The University of Melbourne, Melbourne, VIC 3010, Australia; dtngo@unimelb.edu.au; 3 Karlsruhe Institute of Technology (KIT) Innovation HUB—Prävention Im Bauwesen Ein Helmholtz Innovation Lab, Hermann-von-Helmholtz-Platz 1, 76344 Karslruhe, Germany; andreas.gerdes@kit.edu

**Keywords:** geopolymer concrete, mix design, aluminosilicate precursor, alkaline activator, mechanical properties, theoretical modelling

## Abstract

Geopolymer concrete (GPC) has emerged as a promising low-carbon alternative to ordinary Portland cement (OPC), yet wider adoption is limited by the lack of standardised mix-design procedures. Precursor, activator, curing, and aggregates strongly interact to affect properties, but findings are scattered and hard to generalise. This review consolidates and normalises published findings to clarify how key parameters-precursor type, activator dosage and concentration, activator-to-binder ratio, curing temperature, and aggregate gradation-control fresh and hardened performance. Overall trends indicate that calcium-rich systems enhance early strength by 80–100% at typical replacement levels; while optimum activator conditions of 12 M NaOH and sodium silicate/sodium hydroxide ratio = 2.5 commonly improve strength by 40–60% relative to sub-optimal ratios; alkaline activator-to-binder ratios of 0.4–0.7 provide the most practical strength-workability balance. Heat curing at 80–100 °C significantly accelerates early-age property development by 50–200% compared to ambient curing, depending on duration and activator chemistry. A target-strength mix design is demonstrated through a 40 MPa case study. Using a compiled dataset for fly ash (FA)-based GPC, a modulus–strength framework is proposed; common OPC code equations over-predict elastic modulus for 15–50 MPa, and calibration yields a conservative, code-compatible relation: E = $2.75\sqrt{fc_{MPa}}$. Key limitations are highlighted, including variability in raw materials and durability uncertainties, and future directions are proposed toward performance-based design and standardisation to support structural use of GPC in sustainable infrastructure.

## 1. Introduction

The construction sector remains one of the largest contributors to global greenhouse gas emissions, with OPC alone accounting for nearly 7% of annual CO_2_ output [1]. The energy-intensive clinkerisation process [2], coupled with the rapid growth of infrastructure demand in developing economies, has placed mounting pressure on the industry to adopt lower-carbon alternatives [3,4]. Over the past two decades, GPC has emerged as a viable substitute for OPC-based systems, drawing on industrial by-products such as fly ash (FA), silica fume (SF) [5], ground granulated blast furnace slag (GGBFS) [6], rice husk ash (RHA) [7], ceramic waste powder (CWP), and waste glass powder (WGP) [8] as aluminosilicate precursors. In alkaline environments, these materials undergo dissolution and polycondensation to form sodium/potassium aluminosilicate hydrate (N(K)–A–S–H) or calcium aluminosilicate hydrate (C–A–S–H) gels, eliminating the need for cement clinker and enabling the valorisation of waste streams [9].

**Figure 1:**
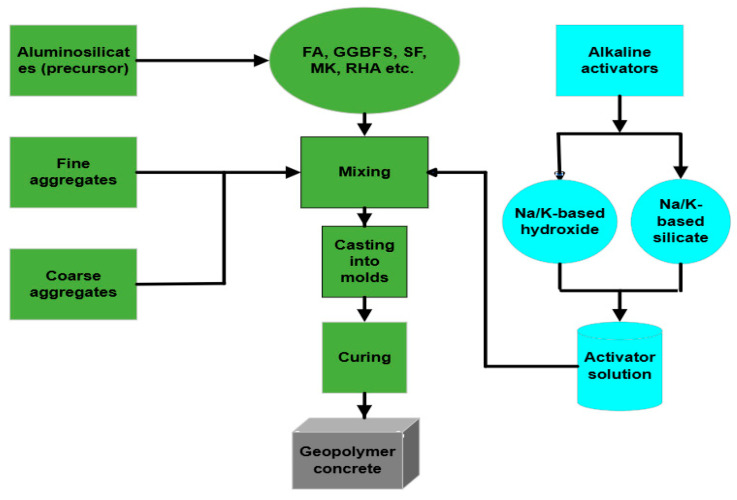
Manufacturing process of GPC, illustrating precursor selection, alkaline activation, mixing, casting, and curing.

GPC has demonstrated feasibility in real projects, including pavement, precast, and marine applications [10,11,12], and several technical specifications have recently emerged (e.g., SA TS 199:2023 [13], ASTM C1928 [14], BSI Flex 350 [15], and Queensland DTMR guidance for precast reinforced GPC [16]). However, large-scale implementation remains constrained, primarily due to the absence of standardised mix-design procedures that can accommodate the variability of precursors and alkaline activator systems. Unlike OPC concrete-where water-to-cement ratio and aggregate gradation provide relatively consistent guidance-GPC performance depends on a broader and strongly coupled parameter set. Precursor chemistry, activator concentration and composition, sodium silicate/sodium hydroxide (SS/SH) ratio, alkali-to-binder (AA/B) ratio, curing regime, and aggregate characteristics collectively govern workability, strength development, and durability. Many studies evaluate these variables in isolation, which contribute to inconsistent conclusions and limit the development of generalisable design rules. The basic procedure adopted by researchers to produce a two-part GPC is given in Figure 1.

**Figure 2:**
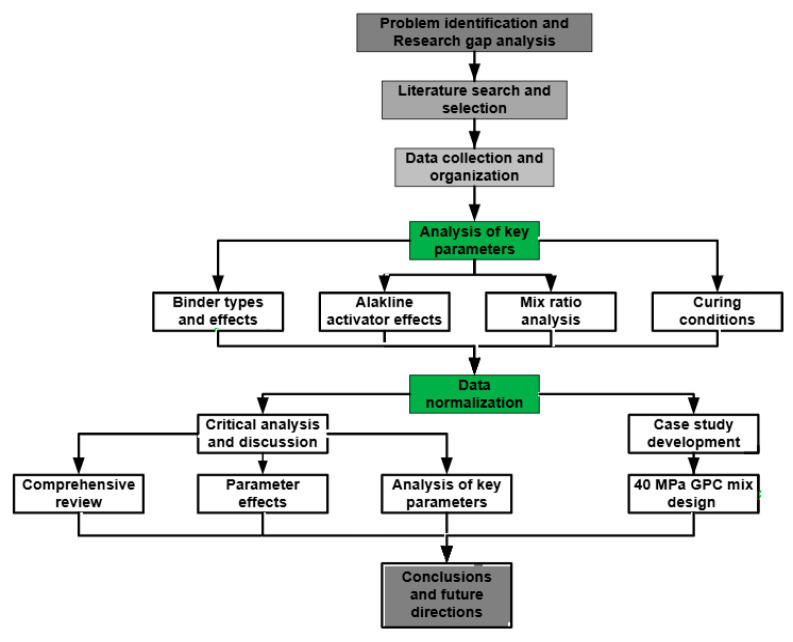
Methodological framework adopted in this review, showing systematic literature evaluation, data normalisation, and synthesis into mixed design recommendations.

Accordingly, this review systematically compiles and normalises published experimental data to quantify the influence of key mix parameters on the fresh and hardened properties of GPC and to identify practical parameter windows for mix design. A target-strength case study (40 MPa) is presented to demonstrate a step-by-step design approach, and a theoretical modulus-strength relationship is proposed to support structural design considerations. Finally, the review highlights current barriers-including raw material variability, curing practicality, and durability data gaps-and outlines priority research directions toward performance-based design and standardisation. The methodological workflow used in this review is summarised in Figure 2.

Literature was retrieved from Scopus, Web of Science, and Google Scholar for the period 2000–2026 using combinations of keywords (‘geopolymer concrete’, ‘alkali activated’, ‘fly ash’, ‘compressive strength’, ‘SS/SH’, ‘molarity’, ‘alkali-to-binder’, ‘curing temperature’, ‘aggregate gradation’). Studies were included if they reported mix proportions and at least one targeted response (slump/workability and/or compressive strength and/or modulus) with identifiable parameter variation. Studies focused on lightweight aggregates, fiber-reinforced systems, or non-concrete geopolymer pastes were excluded unless explicitly used for mechanistic discussion. The scope of this review is limited to fresh properties and hardened mechanical performance, together with a target-strength mix-design demonstration and a modulus-strength model. Durability aspects (e.g., chloride ingress, carbonation, sulfate/acid resistance and long-term cracking) are acknowledged as critical but are not analysed here due to their exposure-specific nature and the need for a dedicated review.

## 2. Binders and Activators Used in GPC Production

The choice of precursor and alkaline activator plays a decisive role in governing the properties of GPC. Industrial and agricultural by-products such as FA, GGBFS, MK, SF, RHA, WGP, and CWP are frequently employed due to their high contents of reactive SiO_2_ and Al_2_O_3_ [17]. These materials serve as the aluminosilicate source for geo-polymerisation, while sodium hydroxide (NaOH) and sodium silicate (Na_2_SiO_3_) solutions are the most adopted activators because of their effectiveness and relatively low cost.

FA remains the most widely used precursor, especially Class F FA, but its limited availability in some regions has prompted increasing reliance on GGBFS and MK. The incorporation of GGBFS improves early strength development owing to the formation of calcium-rich C–A–S–H gels [18], while MK enhances reactivity and matrix densification due to its fine particle size and high alumina content [19]. SF and RHA, despite reducing workability, contribute additional amorphous silica that accelerates gel formation and improves compressive and tensile strength [5,7,20]. In contrast, WGP can adversely affect both workability and strength at high replacement levels, largely because of its angular particle morphology and the risk of alkali–silica reaction [8].

Several studies have investigated combinations of precursors to exploit synergistic effects. For example, FA blended with GGBFS and SF has been shown to yield compressive strengths above 50 MPa while also reducing environmental impacts by up to 60% compared with OPC [6]. High-performance mixes incorporating FA, calcium aluminosilicate cement (CAC), and SF have achieved ultra-high compressive strengths exceeding 130 MPa [20]. Similarly, incorporating RHA with GGBFS and SF has been reported to improve high-temperature resistance and durability [7]. Waste-derived materials such as red mud and olive biomass ash have also been explored as alternative activators, offering cost and CO_2_ reductions. However, mechanical performance is often lower than conventional NaOH–Na_2_SiO_3_ systems [21,22,23].

The use of waste glass-derived sodium silicate has gained recent attention. Vinai et al. [24] and Soutsos et al. [25] demonstrated that sodium silicate powders synthesised from glass cullet can effectively activate FA–GGBFS blends, achieving comparable strength to commercial Na_2_SiO_3_. They further reported compressive strengths of 19 MPa at 60 °C curing when WGP was combined with NaOH as an activator, while Torres-Carrasco and Puertas [26] achieved 40 MPa strength at 85 °C with FA–WGP mixes. Similarly, RHA-derived sodium silicate has been used as a sustainable activator, reducing cost by up to 55% and CO_2_ emissions by 50% relative to commercial sodium silicate [27,28,29].

In summary, the reactivity and performance of GPC are strongly influenced by the chemistry and physical characteristics of the selected precursor and activator. High-calcium binders such as GGBFS provide rapid early strength, whereas MK and SF enhance reactivity and microstructural refinement. Waste-derived activators, particularly those synthesised from glass or biomass ash, show significant promise for improving sustainability and reducing reliance on commercial chemicals, though their variability remains a challenge. A balanced selection of binder and activator, optimised through parameters such as SS/SH ratio, molarity, and AA/B ratio, is therefore essential to achieving desired fresh and hardened properties of GPC. This understanding sets the stage for the parameter-specific evaluations in the next section.

## 3. Effect of Key Influential Parameters on the Properties of Geopolymer Concrete

### 3.1. Effect of Alumina-Silica Source Materials

**Figure 3:**
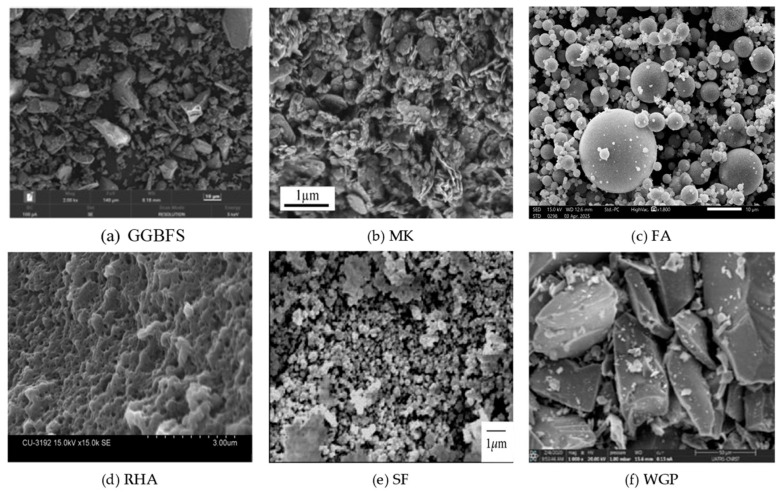
SEM images of major binders used as aluminosilicate source materials in GPC [44,45,46,47,48].

**Figure 4:**
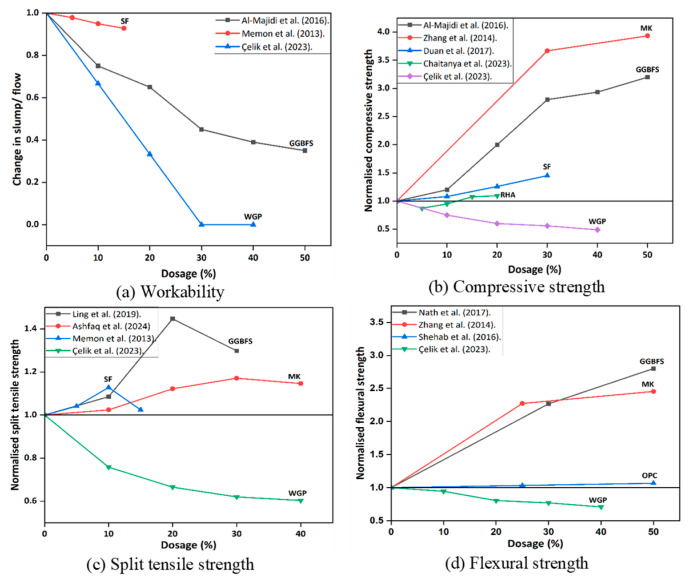
Effect of precursor substitution on the fresh and hardened properties of FA-based GPC (data adopted from [30,31,32,33,34,35,36,37,38,39,41,42]).

The precursor type exerts a dominant influence on both workability and strength of GPC due to variations in chemical composition (Table 1) and particle morphology (Figure 3). Class F FA is widely used. Generally, it requires heat curing to achieve satisfactory strength. Partial replacement with highly reactive materials such as GGBFS, MK, and SF enhances reactivity and early strength development shown in Figure 4.

For instance, incorporating 30–50% GGBFS can increase compressive strength by 80–200% compared with pure FA mixes, owing to the additional C–A–S–H gel formation from calcium-rich slag [30,31,32,33,34,35]. Similarly, MK improves both compressive and flexural strength by up to 250% at 30–40% replacement, producing a denser and more homogeneous microstructure [36]. SF and RHA have also been shown to densify the matrix and raise compressive strength by 45% and 10% when used at 30% and 20% dosage levels, respectively [37,38,39].

However, the addition of these reactive binders typically reduces workability due to their angular particle shape and high surface area. GGBFS can lower the slump by more than 50% at 40% replacement [30,40], while SF and MK cause moderate reductions in flowability [41]. In contrast, WGP sharply decreases workability (up to 67% reduction at 20–40% replacement) [42], and often diminishes strength due to its irregular morphology and the risk of deleterious alkali–silica reactions [42,43]. In conclusion, GGBFS, MK, and SF positively influence strength, while WGP adversely affects both workability and mechanical properties. The statistical trends presented are derived from a compiled dataset of literature mixes, reflecting between-study variability in raw materials, curing and testing protocols. Normalised strength refers to the dimensionless ratio ($X_{norm}$ = $\frac{X}{X_{ref}}$), where X is reported strength (compressive/splitting tensile/flexural) for a given parameter level and $X_{ref}$ is the strength at the defined reference condition within the same study (e.g., baseline mix or optimum/standard condition as specified). This approach preserves within-study trends while reducing between-study variability due to different materials and curing regimes.

**Table 1:** Typical oxide compositions (%) of common precursors used in GPC.

Material	SiO_2_	Al_2_O_3_	CaO	Fe_2_O_3_	MgO	Refs.
OPC	20–25	3–8	60–65	2–6	1–4	[49]
FA	45–60	20–35	5–15	4–10	1–5	
GGBFS	30–40	5–15	30–45	5–10	5–10	
MK	50–60	35–40	<1	<1	<1	
SF	85–95	1–2	<1	<1	<1	
WGP	71.09	3.52	10.59	1.77	1.56	[50]
RHA	82.14	1.34	1.21	1.27	1.96	[51]

### 3.2. Effect of Alakline Activators

#### 3.2.1. Effect of the Sodium Silicate to Sodium Hydroxide Ratio

**Figure 5:**
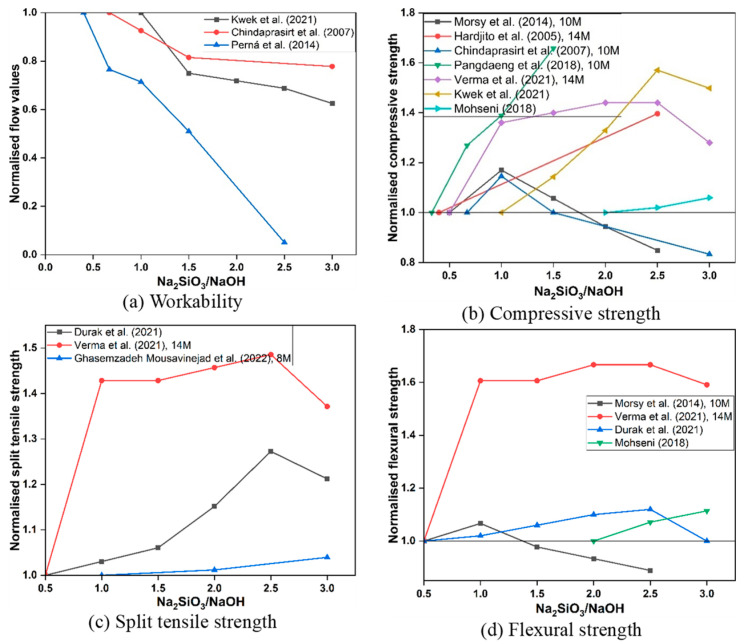
Effect of sodium silicate to sodium hydroxide ratio on the fresh and hardened properties of FA-based GPC (data adopted from [52,53,54,55,57,58]).

The SS/SH ratio governs both the reactivity of the system and the rheological behaviour of the activator, shown in Figure 5. Since SS solutions are significantly more viscous than SH, increasing the SS/SH ratio typically reduces flowability [52]. Reductions of 15–25% in workability have been reported when increasing the ratio from 1.0 to 3.0 [53].

In terms of strength, most studies identify an optimum ratio of 2.5, where compressive strength improvements of 40–60% have been observed relative to lower ratios [54]. At this level, the availability of soluble silicates supports the formation of a dense and homogeneous N–A–S–H gel structure. Strength improvements extend to splitting tensile and flexural performance, with reported gains of 18–70% compared to lower ratios [55]. Beyond the optimum, excess silicate content tends to increase viscosity, hinder dissolution of precursors, and reduce overall gel connectivity, leading to strength losses [53]. While an increase in SS/SH often improves strength by supplying soluble silicate to promote gel formation, several studies reported peculiar decreasing strength at higher SS/SH. These discrepancies can be explained by differences in (i) total alkali dosage (Na_2_O%) and silicate modulus (SiO_2_/Na_2_O). An apparent “high SS/SH” mixture may simultaneously reduce effective NaOH availability, lowering dissolution of FA; (ii) workability and compaction, as higher SS content increases viscosity and can trap air/voids, increasing porosity and reducing strength; and (iii) precursor chemistry, particularly Ca-rich systems where reaction kinetics are already rapid and additional silicate provides diminishing returns or alters gel balance [56]. Therefore, SS/SH effects should be interpreted jointly with Na_2_O dosage, SiO_2_/Na_2_O ratio, and paste rheology rather than as an isolated variable.

#### 3.2.2. Effect of Molarity of Alkaline Activation Solution

**Figure 6:**
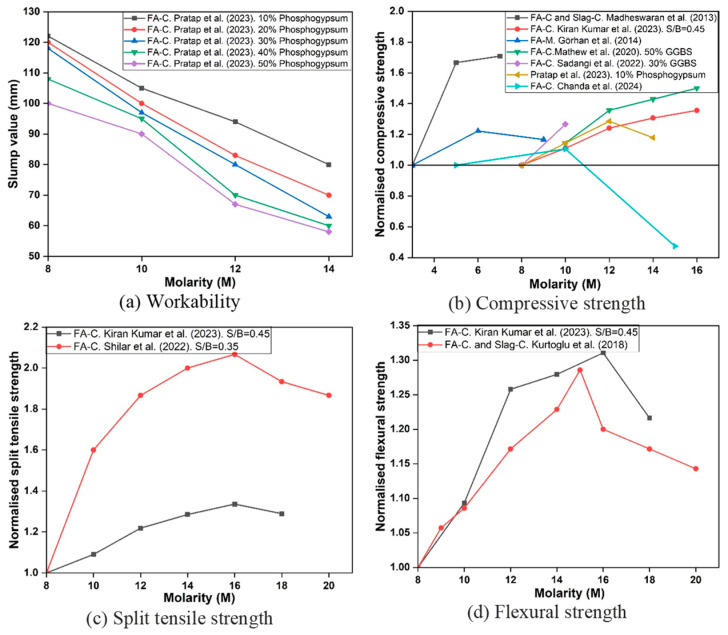
Effect of NaOH molarity on the fresh and hardened properties of GPC (data adopted from [61,64,66,67,68,69,70]).

The molarity of the NaOH solution directly controls the dissolution of aluminosilicate phases and subsequent polycondensation shown in Figure 6. At low molarity (2–6 M), dissolution is incomplete, leading to poor gel formation and low strengths. Increasing molarity to the range of 10–12 M improves dissolution, accelerates gel formation, and enhances both compressive and tensile strength. For example, increases of 20–75% in compressive strength was reported when the molarity was raised from 2 M to 12 M [59].

However, excessively high molarity (>12 M) can have detrimental effects. At very high hydroxide ion concentrations, premature precipitation of aluminosilicate gels occurs, creating a porous matrix and reducing long-term strength [60]. Chanda et al. [61] observed strength reductions of up to 50% when increasing NaOH concentration from 5 M to 15 M. Similarly, porosity and pore size increase at higher molarities, which compromises durability [62,63]. The workability diminishes with elevated molarity because the more viscous nature complicates handling and compaction. At very high molarity, the highly alkaline environment can also increase shrinkage susceptibility and entrapped porosity due to rapid reaction and reduced workability/compaction efficiency, which further reduces compressive strength. The solutions with lower molarity are more manageable and simpler to utilise [61]. Pratap et al. [64] showed a decrease in workability by increasing the molarity level from 8 M to 14 M.

The optimum NaOH molarity varies across studies, and some contradictory trends are expected. In low-Ca FA systems under ambient curing, increasing molarity generally improves dissolution and early strength up to an optimum, after which strength may decline. However, the location of the optimum shifts depending on (i) curing temperature and age, where heat curing can reduce the need for very high molarity; (ii) silicate availability (SS/SH, SiO_2_/Na_2_O), since the combined alkalinity-silicate chemistry controls reaction pathways; and (iii) microstructural side-effects of high molarity, such as rapid precipitation leading to less homogeneous gel, increased shrinkage susceptibility, and reduced workability/compaction quality that increases entrapped porosity. In Ca-rich or hybrid binders, high molarity may be unnecessary or even detrimental due to accelerated setting and microcracking [65]. Accordingly, molarity should be discussed together with precursor Ca-content, curing maturity, and activator modulus rather than treated as a universal single optimum.

#### 3.2.3. Effect of SiO_2_/Na_2_O Ratio

**Figure 7:**
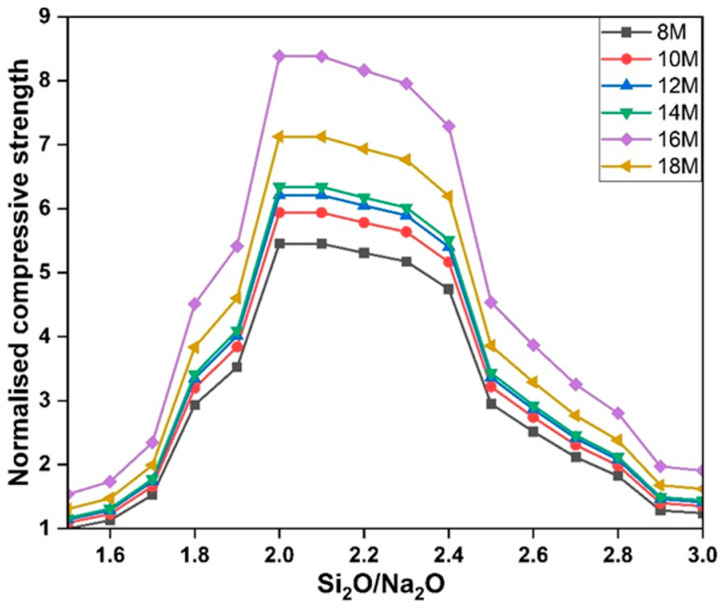
Compressive strengths of GPC as a function of SiO_2_/Na_2_O ratio under different molarity levels (reproduced from [73]).

The silica-to-sodium oxide ratio is a critical indicator of activator chemistry, reflecting the balance between soluble silicates and hydroxide ions. Figure 7 indicates the strength increase with the increase in silica-to-sodium oxide ratio for up to 2 at an SS/SH ratio of 2.5 for 8 M–16 M solutions, with maximum strength achieved using a 16 M solution. At low ratios (<1.0), an excess of OH^−^ promotes rapid precipitation of aluminosilicate gels, resulting in incomplete polymerisation and reduced compressive strength [71]. Increasing the ratio enhances dissolution of precursors and leads to more complex gel networks, improving strength up to an optimum around 2.0–2.5 [72]. Strength gains of up to 60% have been reported when increasing the SiO_2_/Na_2_O ratio from 1.0 to 2.0 [73].

Beyond this optimum, further increases in silicate content raise the viscosity of the activator solution, making mixing and compaction difficult. Excess silicates may also hinder gel connectivity, leading to strength reductions [74]. Overall, an SiO_2_/Na_2_O ratio of 2.0–2.5, when combined with 12 M NaOH and an SS/SH ratio near 2.5, appears to provide the most favourable balance between reactivity, workability, and strength.

#### 3.2.4. Effect of Alkaline Activator/Binder Ratio

**Figure 8:**
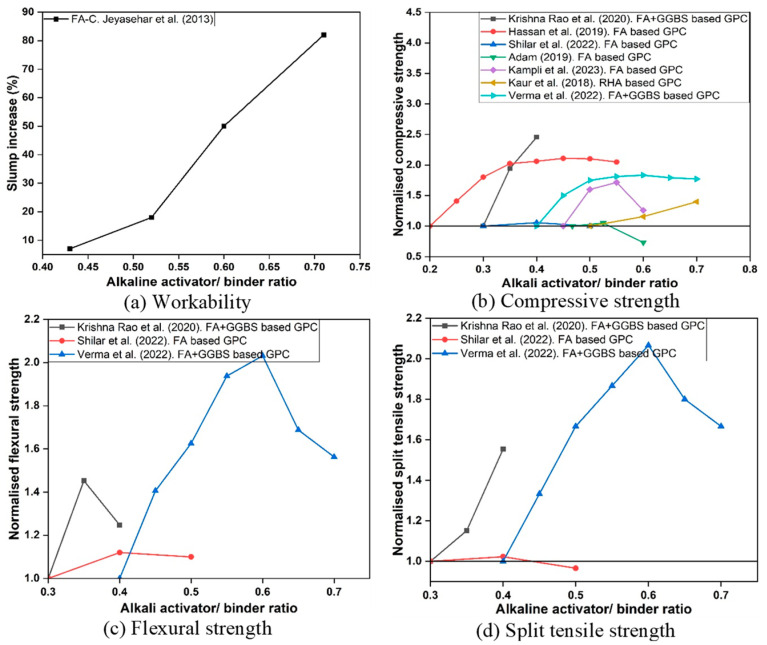
Effect of AA/B ratio on the properties of GPC (data adopted from [39,67,75,76,79,80,81,82]).

The alkaline activator to-binder ratio (AA/B) determines the amount of activator solution available relative to precursor mass, influencing both dissolution kinetics and paste fluidity shown in Figure 8. Increases in AA/B ratio generally enhance workability by increasing liquid content. Reported slump increases of 8–80% have been observed as the ratio rises from 0.43 to 0.71 [75].

Mechanical properties also improve with increasing AA/B ratio up to an optimum of 0.55–0.65. At this range, compressive strength enhancements of 40–100% have been documented compared to lower ratios [39]. Flexural and tensile strengths similarly benefit due to more complete dissolution and gel formation [76]. Microstructural studies confirm that optimal ratios yield denser gel matrices with fewer unreacted precursor particles [77].

However, excessive activator content (>0.7) introduces surplus hydroxide ions, which accelerate premature precipitation and create porosity, thereby reducing strength [78]. Ratios below 0.4, on the other hand, typically lead to poor workability and incomplete geo-polymerisation [67]. Collectively, an AA/B ratio of 0.4–0.7 is recommended for balancing fresh and hardened performance, with system-specific optimisation depending on precursor type. This is because it captures the most frequently reported window where sufficient activator is available for dissolution and gel formation while maintaining workable rheology; AA/B < 0.4 often leads to incomplete reaction and poor workability, whereas AA/B > 0.7 introduces excess liquid/alkali, higher capillary porosity and consequent strength reduction.

### 3.3. Effect of Curing Temperature

**Figure 9:**
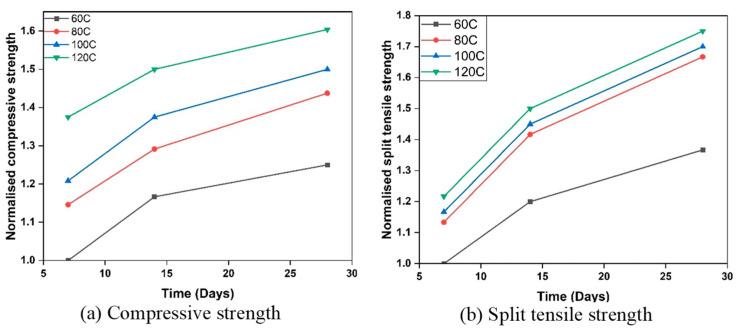
Influence of curing temperature on the mechanical characteristics of GPC [82].

**Figure 10:**
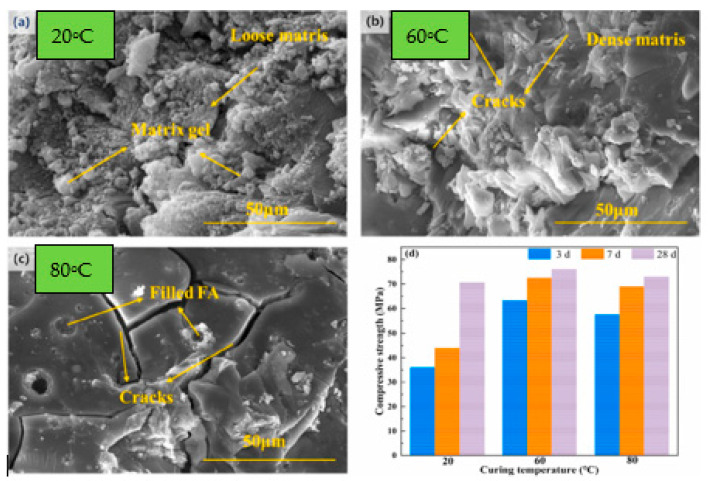
SEM images after curing for 3 days and compressive strength after curing for 3, 7, and 28 days of GP pastes. (**a**) room temperature, (**b**) 60 °C for 24 h, (**c**) 80 °C for 24 h and (**d**) compressive strength (reproduced from [86]).

The curing regime is one of the most influential parameters controlling early-age properties of GPC, shown in Figure 9. Verma et al. [82] explored FA and GGBFS-based concrete by varying curing temperature from 60–120 °C and AA/B ratios from 0.4–0.7. The compressive and split tensile strength increased by 31% and 27% respectively, after 28 days of curing at 120 °C in comparison to 60 °C at an optimum AA/B ratio of 0.6. After 7 days of curing at 120 °C, a significant strength rise of 38% in compressive strength and 21% in tensile strength was seen at an AA/B ratio of 0.6, in comparison to 60 °C. Also, the modulus of elasticity is increased by about 3.8% by curing at 100 °C compared to 60 °C temperature. This is due to the reason, curing temperature accelerates the Geo-P reaction, resulting in a denser and more robust microstructure. Faster reaction kinetics, improved bonding, and reduced porosity at elevated temperatures all contribute to higher mechanical strength and overall durability [83,84]. However, it is important to control the curing temperature carefully, as excessively high temperatures might lead to other issues like rapid drying or thermal cracking [85]. Ma et al. [86] demonstrated a ternary GP from steel slag, FA and GGBS and showed that thermal curing markedly improves early strength shown in Figure 10. Specimens cured at 60 °C for 24 h achieved the highest 3-day compressive strength, followed by 80 °C for 24 h, while room-temperature curing gave the lowest values (Figure 10d). SEM observations indicate that room-temperature sealed curing leads to slow dissolution and polycondensation, producing sparse, loosely packed gels and lower strength (Figure 10a). Curing at 60 °C accelerates reaction kinetics and generates abundant products that fill pores/defects, densifying the matrix and boosting strength (Figure 10b). At 80 °C, rapid free-water loss promotes shrinkage cracking, which compromises microstructural integrity and reduces mechanical performance (Figure 10c).

### 3.4. Effect of Aggregates

**Figure 11:**
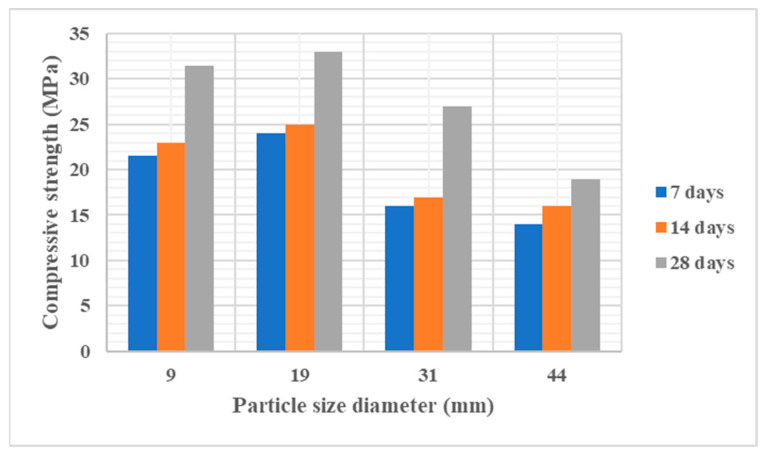
Effect of coarse aggregate size (10–45 mm) on compressive strength of FA-based GPC (reproduced from [91]).

Aggregates typically constitute 60–80% of GPC by volume and thus play a critical role in fresh and hardened behaviour [87], shown in Figure 11. Aggregate properties such as gradation, size, texture, and type influence both workability and strength. Well-graded aggregates improve packing density and reduce porosity, whereas poorly graded systems result in weaker concretes with higher sorptivity [88].

The fine-to-total aggregate ratio is particularly important. Ratios of 0.30–0.40 have been shown to produce optimum strength and workability, with Singh et al. [89] reporting 35% higher compressive strength at a 0.35 ratio compared to mixes with 0.25 or 0.45. Similarly, Pavithra et al. [90] found that maintaining this range improved paste coverage and interfacial bonding.

Aggregate size also exerts a marked effect. Guades [91] demonstrated that 20 mm coarse aggregates achieved the highest compressive strength in FA-based GPC. Larger sizes (>20 mm) can reduce workability and risk segregation, whereas smaller aggregates (<20 mm) require higher paste contents to maintain flowability. Angular aggregates typically enhance mechanical interlocking and strength [92] but reduces workability compared to rounded particles [93].

The use of recycled aggregates is also gaining traction. While untreated recycled aggregates often increase water absorption and reduce strength, surface treatments can mitigate these effects and even enhance bond strength due to additional C–A–S–H gel formation at the interface [94]. In summary, aggregate optimisation is essential for achieving target strength and workability in GPC, with 20 mm maximum size and a fine-to-total ratio of 0.30–0.40 proving most effective across reported studies.

### 3.5. Effect of Chemical Admisxtures and Aggregate Interface Transition Zone (ITZ) Modification

Chemical admixtures and aggregate-binder interfacial transition zone (ITZ) engineering can influence GPC performance. Superplasticizers and viscosity-modifying admixtures may improve dispersion and workability retention in high-alkali mixes; however, their effectiveness is strongly dependent on activator chemistry and precursor composition, and incompatibility may lead to rapid slump loss or abnormal setting [95]. Similarly, ITZ quality governed by aggregate packing, paste volume, and surface reactivity controls crack initiation and stress transfer; strategies such as optimised grading, incorporation of reactive micro-fillers (e.g., silica-rich fines), and surface treatments can densify the ITZ and reduce porosity, improving strength and stiffness [96].

In summary, the performance of GPC is highly sensitive to variations in mix design parameters, with each factor, whether chemical or physical, playing a pivotal role. Optimising precursor selection, activator ratios, molarity, and curing conditions within identified thresholds enables the development of high-performance, durable, and workable GPC. These insights form the basis for rational mix design strategies, as outlined in the following section.

## 4. Mix Design of Geopolymer Concrete

The mix design of GPC remains a major challenge because the conventional procedures established for OPC concrete, such as the water-to-cement ratio approach in ACI 211.1 [97], cannot be directly applied. Unlike OPC systems, GPC performance is controlled by a broader set of interacting parameters, including precursor chemistry, activator molarity and composition, alkali-to-binder ratio, curing conditions, and aggregate volume. As a result, identical mix proportions may yield significantly different outcomes when applied to different raw materials [98]. To address this complexity, three principal approaches have been proposed: the target strength method (TSM), the performance-based method (PBM), and statistical or empirical methods (SM).

Target Strength Method (TSM): The TSM adapts the OPC design philosophy by selecting mix parameters to achieve a specified compressive strength. Researchers adjust the AA/B ratio and activator content based on target performance, then calculate binder and aggregate proportions accordingly. Pavithra et al. [90] and Reddy et al. [99] demonstrated that this method provides practical guidance for structural GPC, establishing correlations between AA/B ratio and compressive strength comparable to the W/B ratio curves for OPC. Ferdous et al. [87] fixed the binder content and varied the concrete densities, specific gravity of ingredients, and air volume. However, TSM focuses narrowly on compressive strength and may neglect durability and workability requirements.

Performance-Based Method (PBM): PBM extends beyond strength to incorporate durability indicators such as chloride penetration, sorptivity, and permeability. Noushini and Castel [100] applied PBM for marine GPC, showing that conventional strength-based indicators may not reliably predict chloride resistance. Similarly, Bondar et al. [101] considered slump and chloride diffusivity as coequal performance criteria. Although PBM better reflects long-term serviceability, it requires more extensive testing and is not yet widely standardised.

Statistical and Empirical Methods (SM): Several studies have employed regression models, response surface methodology, and Taguchi design of experiments to capture the relative influence of parameters such as molarity, SS/SH ratio, and curing regime [102]. While these methods provide insights into parameter interactions, their applicability is often restricted to the datasets from which they are derived.

**Figure 12:**
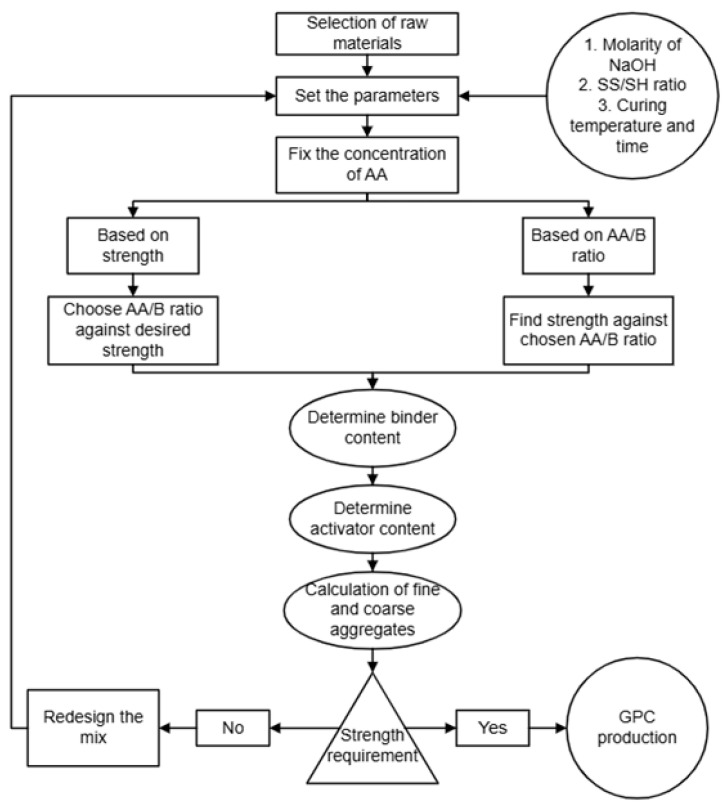
Flow chart for the mix design of GPC.

Figure 12 shows the flow chart considered by researchers for developing a mixed design of GPC [90,103]. In the following sub-sections, an attempt is made at the mix design of FA-based and GGBFS-based GPC following the TSM.

It is important, therefore, to have a better understanding of the properties of thermoplastic composites with fillers and manufactured using different processes.

A target compressive strength of 40 MPa was adopted as a representative normal-strength structural concrete grade commonly specified in practice for general reinforced concrete applications. This level also lies within the most frequently reported performance range for FA-based and blended GPC under ambient-to-moderate thermal curing, enabling the case study to be developed using a broad and reliable literature base without extrapolation. The proposed target-strength framework is generic and can be readily extended to other strength grades according to project requirements.

### 4.1. Alumina-Silica Source Material

The selection of appropriate Al-Si source materials represents the foundational step in designing GPC for a target strength of 40 MPa. When selecting precursor materials: (1) the ratio of SiO_2_ to Al_2_O_3_ should ideally fall between 2.0 and 3.5 [65], (2) CaO content influences setting time and early strength development [65], (3) Fe_2_O_3_ content should be monitored as it can affect workability [104]. The precursor systems used in this study are FA, GGBFS, FA–RHA, and FA–SF.

### 4.2. Alkaline Activator/Binder Ratio

**Figure 13:**
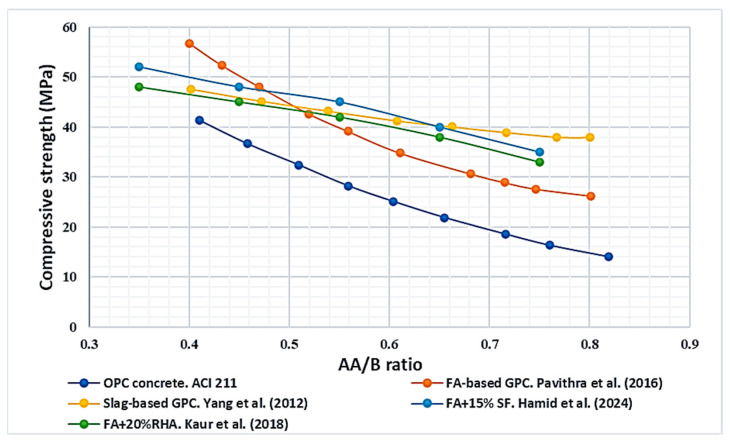
Compressive strength as a function of AA/B ratio for GPC and W/B ratio for OPC concrete (data adopted from [5,39,90,97,105].

The researchers correlated the alkaline activator to binder ratio (AA/B) of GPC with the water/binder ratio of OPCC by following the ACI 211.1 [97]. The strength of GPC and AA/B correlation was calculated and compared to the ACI curve shown in Figure 13. Like OPC concrete, GPC reveals a decrease in strength with a rise in AA/B ratio above the optimum limit. The compressive strength of GPC often exceeds that of OPCC at equivalent AA/B or W/B ratios, demonstrating the superior mechanical properties of GPC. By following the Pavithra et al. [90], Yang et al. [105], Kaur et al. [39], Hamed et al. [5] the AA/B ratios for FA, GGBFS, FA-RHA, and FA-SF-based GPC corresponding to the 40 MPa strength are 0.55, 0.68, 0.60, and 0.65, respectively.

### 4.3. Alkaline Activator and Binder Content

The quantity of alkaline activator and binder was determined by adapting the water content selection procedure in ACI 211.1 [97] to GPC systems. In conventional OPC mix design, the required water content is selected based on the target slump and nominal maximum aggregate size. In this study, the same philosophy was adopted, but the “water” term was replaced by the mass of alkaline activator solution (AA), which represents the combined mass of NaOH solution (SH) and sodium silicate solution (SS). Published data for FA-, GGBFS-, FA–RHA-, and FA–SF-based GPC [5,39,97,105,106] were used to calibrate activator requirements for different slump ranges and aggregate sizes, as summarised in Table 2, Table 3 and Table 4.

For the 40 MPa target-strength case study, a nominal maximum aggregate size of 19–20 mm and a slump of 150–200 mm was selected, consistent with typical commercial ready-mixed concrete. From Table 3, Table 4 and Table 5, the corresponding activator requirements (AA) were taken as 216, 215, 216, and 222 kg/m^3^ for FA-based, GGBFS-based, FA–RHA-based, and FA–SF-based GPC, respectively. These values are analogous to the “water content” in OPC mix design, but here they represent the total mass of the alkaline activator solution. The next step is to split this total activator mass into NaOH (SH) and sodium silicate (SS) based on the selected SS/SH ratio. A ratio of 2.0 was adopted in all mixes, as this value has been widely reported to provide a good compromise between workability, strength, and cost. For a given total activator mass (SS + SH), the individual components are obtained from: SS/SH = 2.0 and SS + SH = AA.

Solving these equations gives SH = AA/3 and SS = 2AA/3. As an example, for FA-based GPC with AA = 216 kg/m^3^:

SH = 216/3 = 72 kg/m^3^, SS = 2 × 216/3 = 144 kg/m^3^.

An identical procedure is followed for the other systems, yielding SH = 71.7 and SS = 143.3 kg/m^3^ for GGBFS-based GPC, SH = 72 and SS = 144 kg/m^3^ for FA–RHA-based GPC, and SH = 74 and SS = 148 kg/m^3^ for FA–SF-based GPC.

Once the activator content is fixed, the corresponding binder (precursor) content is obtained from the selected alkali-to-binder (AA/B) ratios associated with the 40 MPa strength level. Based on correlations reported by Pavithra et al. [80], Yang et al. [93], Kaur et al. [39] and Hamed et al. [5], AA/B ratios of 0.55, 0.68, 0.60, and 0.65 were adopted for FA-, GGBFS-, FA-RHA-, and FA-SF-based GPC, respectively. Rearranging AA/B = AA/Binder gives:

Binder = AA/(AA/B)

For instance, for FA-based GPC:

AA/B = 0.55

Binder (FA) = 216/0.55 = 393 kg/m^3^

For GGBFS-based GPC:

AA/B = 0.68

Binder (GGBFS) = 215/0.68 = 317 kg/m^3^

For the blended FA–RHA system, the total binder mass is first calculated using AA/B = 0.60:

Binder (FA + RHA) = 216/0.60 = 360 kg/m^3^

The individual precursor masses are then obtained from the specified 80:20 FA:RHA ratio, giving FA = 288 kg/m^3^ and RHA = 72 kg/m^3^. A similar approach is used for the FA–SF blend: with AA = 222 kg/m^3^ and AA/B = 0.65, the total binder mass is 342 kg/m^3^, which is then partitioned into FA (290.7 kg/m^3^) and SF (51.3 kg/m^3^) based on the adopted 85:15 ratio.

It is important to note that precursor fineness also influences the required binder content at a given strength. The higher FA fineness leads to increased strength at the same binder dosage. For the present case study, an FA fineness of 440 m^2^/kg was considered; according to Patankar et al. [96], this fineness level requires approximately 430 kg/m^3^ of FA to achieve 40 MPa, which is slightly higher than the value back calculated from the AA/B ratio alone. Such observations emphasise that the AA/B-based design should be cross-checked against fineness-dependent strength-binder correlations to ensure that the selected binder content is realistic.

### 4.4. Quantity of Aggregates

The aggregate content was determined by extending conventional OPC mix design principles to GPC. Like OPC concrete, the aggregate skeleton in GPC governs packing density, workability, and mechanical performance [108]. Most studies on normal-weight GPC report total aggregate volumes in the range of 60–80% of the concrete volume [109]. In this case study, a total aggregate volume fraction of 60% was adopted as a representative value for structural GPC.

The procedure for determining aggregate quantities involves three main steps: (i) selecting a suitable binder-to-sand (fine aggregate) ratio, (ii) fixing the fine-to-total aggregate ratio, and (iii) converting the resulting volumes to masses using typical aggregate densities. In both OPC and GPC, binder-to-sand ratios between 1:1.5 and 1:2.5 are commonly used. For FA-based GPC in this study, a binder-to-sand ratio of 1:2 was chosen, which yields a sand content equal to twice the binder mass. With a binder mass of 393 kg/m^3^, this results in a sand requirement of 786 kg/m^3^. Corresponding sand contents for the GGBFS-, FA–RHA-, and FA–SF-based mixes were obtained in the same way, as reported in Table 5.

**Figure 14:**
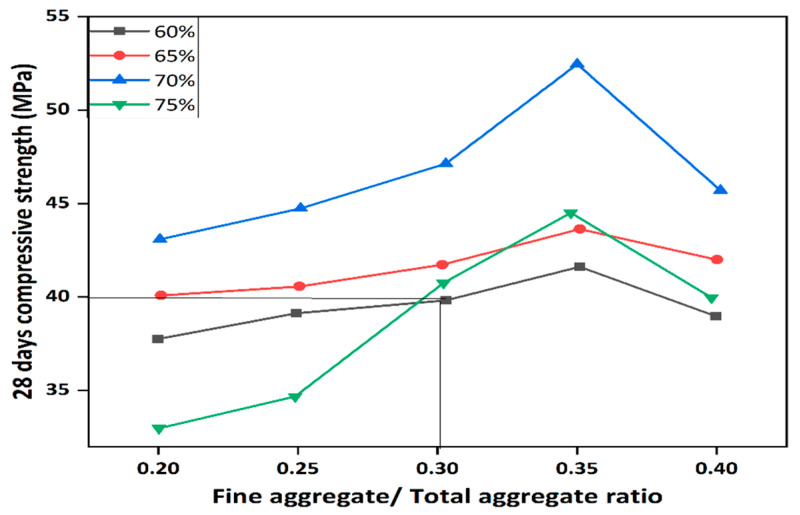
Compressive strength as a function of fine to coarse aggregates for different volumes of aggregates [109].

Next, the fine-to-total aggregate ratio (F.A/Total aggregate) must be selected. Experimental work by Joseph and Mathew [109] shown in Figure 14, showed that for total aggregate contents of 60–75% by volume, a fine-to-total aggregate ratio in the range 0.30–0.40 provides a good balance between workability and strength. In this study, a ratio of 0.30 was adopted for the 40 MPa target-strength mixes, consistent with the optimum observed for 60% total aggregate volume.

Once the fine-to-total aggregate ratio is fixed, the total aggregate mass can be calculated from the sand mass:

F.A/Total aggregate = 0.30 → Total aggregate = F.A/0.30

The coarse aggregate (C.A) content is then simply:

C.A = Total aggregate − F.A

This procedure was followed for each binder system, and the final sand and coarse aggregate quantities per cubic metre are summarised in Table 6. Overall, this approach ensures that the aggregate skeleton in GPC is designed using a framework analogous to OPC mix design, but with parameters calibrated to geopolymer systems. The integration of AA/B-based binder selection, fineness-sensitive binder requirements, and aggregate optimisation provides a rational, target-strength mix design methodology for 40 MPa GPC incorporating different precursor systems.

## 5. Theoretical Modelling (Compressive Strength-Modulus Relationship)

### 5.1. Aim and Scope

This section establishes an empirical relationship between compressive strength (fc) and modulus of elasticity (E) for FA–based GPC. We collected paired values (fc, E) from the literature and benchmark the trends against OPC code expressions and GPC-specific correlations [31,110,111,112,113,114,115,116,117,118,119]. Only normal-weight concretes were considered; lightweight and fibre-reinforced mixes were excluded. In concrete-like composites, the elastic modulus reflects the combined stiffness of the aggregate skeleton, the binder matrix, and the interfacial transition zone (ITZ). For FA-GPC, increases in fc′ generally correspond to increased gel formation and reduced porosity, which stiffen the matrix and improve ITZ load transfer; however, *E* is also highly sensitive to aggregate stiffness and density, as well as microcracking/shrinkage. Therefore, compared to OPC, FA-GPC can exhibit lower modulus at similar fc′ due to differences in gel structure, pore connectivity, and ITZ characteristics, motivating a dedicated FA-GPC correlation rather than direct use of OPC-based code expressions. The objective is (i) to show how FA-GPC compares to conventional OPC predictions and (ii) to derive a suitable GPC equation for design-level estimates within the studied ranges.

### 5.2. Methodology

To benchmark GPC against established practice, ordinary Portland cement concrete (OPCC) relations (Equations (1)–(4)) were plotted on the same axes along with two concise literature relations (Equations (5) and (6)) commonly reported for FA-GPC as reference curves.
ACI 318: [120]$E_{ACI318}=4700\sqrt{fc}\;\;\left(\mathrm{MPa}\right)$Equation (1)AS 3600: [121]$E_{\rho }=\rho^{1.5}\cdot \;\left(0.024\sqrt{fc}+0.12\right)\;\left(\mathrm{MPa}\right)$Equation (2)ACI 363R:$E_{ACI363}=3320\sqrt{fc}+6900\;\;\left(\mathrm{MPa}\right)$Equation (3)Eurocode 2: [122]$E_{Eurocode\;2}=22{\left(\frac{fcm}{10}\right)}^{0.3}\;\;\left(\mathrm{GPa}\right),\;fcm=fc+8\mathrm{MPa}$Equation (4)Nath & Sarker: [31]$E_{N\&S}=3510\sqrt{fc}\;\;\left(\mathrm{MPa}\right)$Equation (5)Noushini et al. [100]$E_{N}=-11400+4712\sqrt{fcm}\;\;\left(\mathrm{MPa}\right)$Equation (6)

Model confidence was assessed by external benchmarking against commonly used OPC code relations (ACI 318, AS/ACI density form, ACI 363R, Eurocode 2) and published FA-GPC correlations (e.g., Nath & Sarker; Noushini).

### 5.3. Results and Analysis

**Figure 15:**
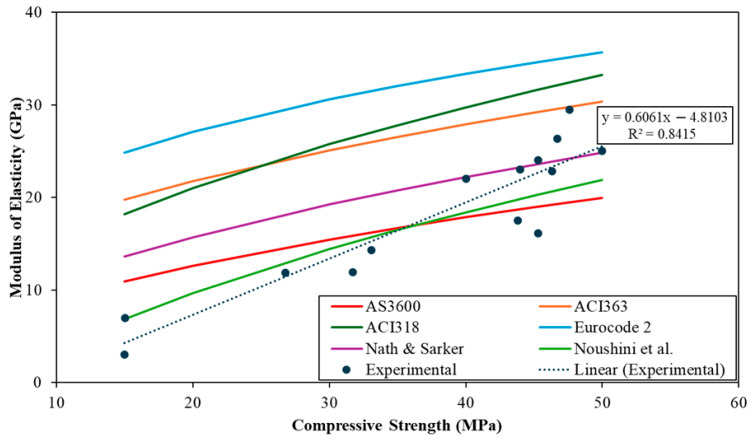
Modulus of elasticity vs. compressive strength for OPC references and FA-based GPC (data adopted from [31,100,120,121,122]).

Figure 15 compares the compiled FA-GPC measurements with OPC reference curves (AS 3600/ACI density form, ACI 318, ACI 363R, Eurocode 2) and two GPC-specific relations (Nath & Sarker; Noushini). Across 15–50 MPa the FA-GPC points remain consistently below the OPC code lines, with Eurocode 2 forming the upper envelope and ACI 318/ACI 363R also over-predicting. In contrast, the Nath–Sarker and Noushini curves follow the experimental values more closely, confirming the lower stiffness coefficient.

**Figure 16:**
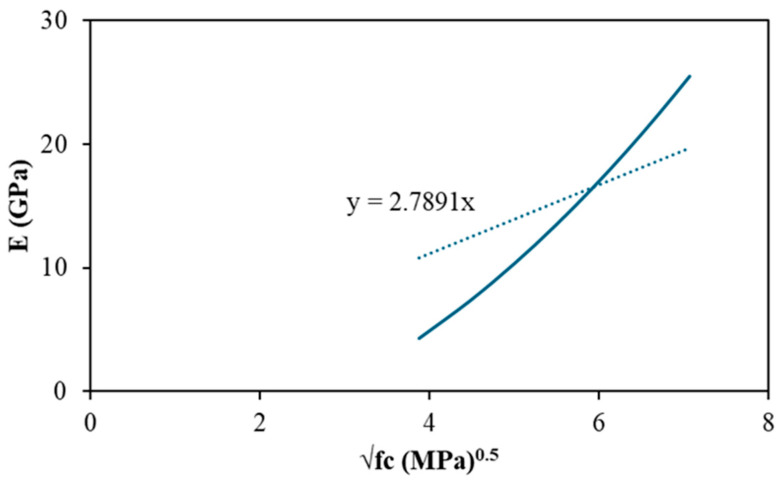
Relationship between elastic modulus and compressive strength for FA-based geopolymer concrete compiled from the literature (data points extracted from Refs. [31,110,111,112,113,114,115,116,117,118,119].

The fitting of present experimental dataset gives a strong linear trend (dotted line in Figure 16) shown in Equation (7).

(7) $$E_{GPa}=0.6061fc\;\left(\mathrm{MPa}\right)-4.8103;R^{2}=0.84$$

which yields representative predictions of E = 13.4 GPa at fc = 30 MPa, 19.4 GPa at 40 MPa, and 25.5 GPa at 50 MPa. These values sit within the range of FA-GPC measurements and below OPC references over the plotted range.

Because most design provisions express modulus as a function of √fc, a code-compatible form (E = k√fc) was also calibrated to the same data. The coefficient k was obtained using two approaches. (i) by plotting modulus E versus √fc (shown in Figure 16). The slope of line, taking the intercept 0, gives the value of k, and (ii) range-matched calibration (least squares over the chosen strength range (15–50 MPa)) using Equation (8) [123].

The fitted curve(s) and proposed equation are developed in this study based on regression of the compiled dataset.

(8) $$k=\frac{\int_{15}^{50}0.6061fc\;\left(\mathrm{MPa}\right)-4.8103)\sqrt{fc}\;df}{\int_{15}^{50}\left(f\right)\;df}=2.75\mathrm{GPa}.{\;\mathrm{MPa}}^{-0.5}$$

The resulting relation (Equation (9)) reproduces the central tendency of the FA-GPC data.

(9) $$E_{GPa}=2.75\sqrt{fc_{MPa}}$$

Here E is the etatic modulus of elasticity (GPa) and fc’ is the 28-day compressive strength (MPa); $\sqrt{fc_{MPa}}$ denotes the square root of compressive strength.

The regression in Figure 16 provides a quantitative measure of the strength-stiffness relationship for the compiled FA-GPC dataset. The fitted trend yields a correlation coefficient of R^2^ = 0.8415, indicating that approximately 84% of the variation in elastic modulus is explained by compressive strength within the analysed range (15–50 MPa). This level of agreement supports the use of the proposed equation as a design-level predictor for normal-weight FA-GPC. The remaining scatter is expected for a literature-compiled database because E is sensitive to between-study differences in precursor chemistry, curing regime, aggregate stiffness/density, and the modulus testing definition (e.g., secant modulus level). Accordingly, the proposed relation should be applied within the stated range and interpreted as a conservative, practical correlation rather than a universal constitutive law.

The comparison of elastic modulus predictions at representative compressive strengths against GPC-specific relations and OPC baselines is illustrated in Table 6. OPC code relations over-predict modulus (+55–165%), whereas GPC-specific equations are much closer but still above line (+5–28%).

The proposed relation is intended as a design-level predictor for FA-GPC within the stated range; variability across studies (precursor chemistry, curing history, aggregate type/density, and modulus test definition) contributes to scatter and may require recalibration for other precursor systems.

## 6. Conclusions

This review consolidates and normalises published evidence to translate the fragmented geopolymer concrete (GPC) literature into practical mix-design principles. Overall, GPC performance is governed by the coupled control of (i) reactive binder chemistry (Ca availability and amorphous content), (ii) activator alkalinity and silicate supply (NaOH concentration and SS/SH balance), (iii) liquid dosage (AA/B) that determines dissolution and pore structure, and (iv) curing maturity that drives reaction extent-none of which should be optimised in isolation. Based on the synthesised trends, strength gain is most effectively achieved by increasing reactive Ca (e.g., GGBFS blending) while maintaining workable rheology, and by adopting moderate activator conditions (SS/SH around 2.5 and NaOH around 12 M for FA systems) to avoid excessive viscosity, rapid precipitation, and porosity that reduce strength at high molarity. The preferred AA/B window (0.4–0.7) reflects the practical balance between sufficient alkalinity/gel formation and avoidance of excess free liquid and capillary voids.

From an engineering standpoint, the 40 MPa case study demonstrates how these principles can be operationalised into a target-strength workflow for common precursor systems (FA-, slag- and hybrid-based mixes). For structural design support, the compiled FA-GPC dataset confirms that OPC-based modulus expressions are unconservative for GPC and supports a conservative, code-compatible stiffness predictor E = $2.75\sqrt{fc_{MPa}}$ within 15–50 MPa for normal-weight, predominantly ambient-cured FA-GPC.

Future research directions and practical implementation priorities

Future work should prioritise (i) short-term constructability and QA/QC, including standardised reporting of precursor/activator chemistry, workability retention and setting control, and simple field acceptance tests; (ii) medium-term durability-linked mix design, quantifying how AA/B, molarity, SS/SH, curing regime and Ca-content govern transport, shrinkage/cracking, carbonation and chloride resistance; and (iii) long-term standardisation and code integration, supported by structural-scale validation and supply-chain strategies to manage precursor variability. Although durability is essential for structural applications, a full durability synthesis is outside the scope of this paper. Future work should explicitly link key mix parameters (precursor chemistry, activator dosage/modulus, AA/B ratio and curing maturity) to durability indicators such as transport properties, cracking/shrinkage susceptibility and chemical resistance under defined exposure classes. Practical deployment challenges-such as activator handling and safety, curing logistics, batch-to-batch variability, and site quality control-should be addressed alongside material optimisation to enable reliable large-scale applications.

Overall, this review provides a unified synthesis and practical guidance for developing standardised GPC mix design procedures, thereby supporting its broader implementation in structural and sustainable infrastructure applications.

## Figures and Tables

**Table 2:** ACI 211.1 water and air content selection as a function of slump and nominal maximum coarse aggregate size (mm) (9.5–150 mm) used here as an analogue for activator selection in FA-based GPC [98].

Slump (mm)	Water Requirement (kg/m^3^) for Nominal Mean Size Aggregate (mm)							
	9.5	12.5	**19**	25	37.5	50	75	150
25–50	207	199	190	179	166	154	130	113
75–100	228	216	205	193	181	169	145	124
**150–175**	243	228	**216**	202	190	178	160	-
Entrapped air (%)	3	2.5	2	1.5	1	0.5	0.3	0.2

*Note: Bold values indicate the parameters selected for the case study.*

**Table 3:** Approximate amount of water selection for slumps with varied nominal mean size aggregate for GGBFS-based GPC (adapted from [105]).

Slump (mm)	Water Requirement (kg/m^3^) for Nominal Mean Size Aggregate (mm)							
	Pebble stone				Crushed stone			
	5	10	20	40	5	10	**20**	40
10–30	220	190	165	150	230	200	180	170
50–70	230	205	180	170	240	215	195	185
100–120	235	215	195	180	250	225	203	195
**150–200**	245	220	205	190	260	235	**215**	205

*Note: Bold values indicate the parameters selected for the case study.*

**Table 4:** Approximate amount of alkaline activator requirement for slumps with varied nominal mean size aggregate for FA-RHA-based and FA-SF-based GPC (reproduced from [106,107]).

Mix Type	Slump (mm)	Alkaline Activator Requirement (kg/m^3^) for Nominal Mean Size Aggregate (mm)			
		9.5	12.5	**19**	25
FA-RHA (80:20)	25–50	207	199	190	179
	75–100	228	216	205	193
	**150–175**	243	228	**216**	202
FA-SF (85:15)	25–50	215	205	195	185
	75–100	235	225	212	198
	**150–175**	250	235	**222**	208

*Note: Column headers (9.5, 12.5, 19 and 25) denote nominal maximum coarse aggregate size (mm). Bold values indicate the parameters selected for the case study.*

**Table 5:** Quantities of the ingredients required to make 40 MPa FA-based, GGBFS-based, FA-RHA-based, and FA-SF-based GPC.

Parameter	FA-Based	GGBFS-Based	FA-RHA-Based	FA-SF-Based	Measuring Unit
AA/B ratio	0.55	0.68	0.60	0.65	-
AA	216	215	216	222	kg/m^3^
SH	72	71.66	72	74	kg/m^3^
SS	144	143.34	144	148	kg/m^3^
Precursor	393	317	288 + 72	290.7 + 51.3	kg/m^3^
Sand	786	634	720	684	kg/m^3^
C.A	2620	2113	2400	2280	kg/m^3^

Nominal mean aggregate size of 20 mm, slump of 150–200 mm, molarity of SH is 10 M, SS/SH ratio of 2, precursor to sand ratio of 1:2, and aggregates volume 60% of the total matrix. Coarse aggregates (C.A).

**Table 6:** Pointwise comparison of elastic modulus predictions at representative compressive strengths.

fc′ (MPa)	Elastic Modulus (GPa)						
	P	N & S	Noushni	ACI318	AS3600	ACI363	EC2
15	10.7	13.6 (27)	11.2 (5)	18.2 (71)	19.6 (84)	19.8 (85)	28.3 (165)
30	15.1	19.2 (27)	17.7 (17)	25.7 (71)	27.7 (84)	25.1 (66)	32.8 (118)
40	17.9	22.2 (24)	21.3 (18)	29.7 (66)	32.0 (79)	27.9 (56)	35.2 (97)
50	19.5	24.8 (27)	24.5 (26)	33.4 (71)	35.8 (84)	30.4 (56)	37.3 (92)

Note: The values in parentheses indicate % higher than the proposed line. fc in MPa, Proposed (P), Nath and Sarker (N & S), and Eurocode 2 (EC2).

## Data Availability

Data will be made available on request.
